# The race against time: patterns and variables of spine surgery timing in traumatic spinal cord injury: a retrospective cohort study from the TraumaRegister DGU®

**DOI:** 10.1186/s42466-025-00429-5

**Published:** 2025-10-10

**Authors:** Till Kamradt, Stefan Hemmer, Raphael Trefzer, Gerhard Schmidmaier, Andreas Hug, Rüdiger Rupp, Rolf Lefering, Norbert Weidner

**Affiliations:** 1 Spinal Cord Injury Center, Heidelberg University Hospital, Schlierbacher Landstrasse 200a, 691118 Heidelberg, Germany; 2 Clinic for Orthopedics, Heidelberg University Hospital, Heidelberg, Germany; 3 Clinic for Trauma Surgery, Heidelberg University Hospital, Heidelberg, Germany; 4 Medical Faculty Heidelberg, Heidelberg University, Heidelberg, Germany; 5 Faculty of Health, IFOM – Institute for Research in Operative Medicine, University Witten/Herdecke, Cologne, Germany

**Keywords:** Acute spinal cord injury, Traumatic, Spine surgery, Decompression, Functional impairment, Confounding factors

## Abstract

**Background:**

Numerous uncontrolled observational studies suggest that early spinal decompression and stabilization within 24 h of spinal cord injury (SCI) improve neurological recovery, forming the basis for recently published best practice guidelines. In this study, we aim to investigate current surgical practices in trauma centers across Germany, Austria, and Switzerland and to elucidate trauma- and patient-related factors influencing the timing of spine surgery.

**Methods:**

We identified patients aged 16 years or older with traumatic SCI and permanent neurological deficits from the TraumaRegister DGU^®^ of the German Trauma Society (2008–2022). Trauma severity was assessed using the Abbreviated Injury Scale. Patients were categorized based on the timing of spine surgery (early surgery: day of admission; late surgery: subsequent days) and functional impairment (moderate vs. severe, based on the Glasgow Outcome Scale). Multivariate regression analyses were conducted to correlate patient and trauma-related factors with these endpoints.

**Results:**

A total of 9938 patients with SCI at cervical, thoracic, and lumbar levels were identified. Among the 5025 patients who underwent spine surgery, 69% were operated on the day of admission, while 31% received surgery on subsequent days. Elderly patients (≥ 60 years) had a higher likelihood of delayed surgery (odds ratio [OR] 0.68–0.76). Trauma-related factors, including high cervical SCI, significant injuries beyond the spine, traumatic brain injury, and signs of hemorrhage, were strongly associated with late surgery (OR 0.38–0.83; *p* < 0.05). Conversely, patients with complete SCI or SCI at the thoracic or lumbar levels were more likely to undergo early surgery (OR 1.45–1.8; *p* < 0.001). Severe functional impairment was associated with advanced age (≥ 70 years), complete SCI, high cervical SCI, concomitant traumatic brain, signs of hemorrhage and comorbidities (OR 1.27–4.59; *p* < 0.01), whereas SCI at thoracic (OR 0.8) and lumbar (OR 0.4) levels correlated with moderate functional impairment (*p* < 0.01).

**Conclusion:**

The majority of SCI patients in trauma centers across Germany, Austria, and Switzerland undergo early spinal surgery, reflecting adherence to best practice recommendations. Timing of surgery is significantly influenced by patient age and trauma complexity. Delays are more common in elderly patients and those with high cervical injuries or associated trauma, underscoring the need for individualized surgical decision-making. Given the strong correlation between injury severity, surgical timing, and functional impairment, future guidelines should refine criteria for early intervention to further optimize neurological recovery.

**Supplementary Information:**

The online version contains supplementary material available at 10.1186/s42466-025-00429-5.

## Background

Traumatic spinal cord injury (SCI) is characterized by a highly localized lesion within the central nervous system, leading to neurological and functional impairment primarily defined by the severity and level of injury. State-of-the-art treatments worldwide include spine surgery (decompression and stabilization) followed by comprehensive care at specialized SCI centers. The goal is to maximize functional recovery and independence through individualized rehabilitative interventions while preventing or managing secondary complications such as pressure sores, bacterial infections, pain, and spasticity [24].

The trauma-induced impact on the spinal cord, along with spinal canal narrowing and spinal cord edema, increases intraparenchymal pressure, which negatively affects spinal cord perfusion and contributes to secondary damage [16]. Spinal cord decompression surgery aims to reduce intraparenchymal pressure and improve spinal cord perfusion, ultimately limiting neurological impairment [21].

Several non-randomized observational studies suggest—but do not confirm—that early decompression surgery within 12–24 h post-injury may improve neurological recovery [4, 7]. However, this remains debated, as other studies have found no significant neurological advantage of early decompression surgery [11, 23]. A major limitation of observational studies without proper control groups is that the timing of surgery is influenced by confounding factors, which may significantly affect neurological and functional outcomes. A recent prospective multicenter study involving 294 acute traumatic SCI patients across Europe underscored the importance of adjusting for confounding factors when analyzing neurological and functional outcomes [11]. Despite of these limitations early spine surgery meaning spinal cord decompression within 24 h is recommended according to a recently published best practice guideline in Germany [6].

The present study aims to comprehensively analyze current spine surgery practices following SCI in acute care hospitals across Germany, Austria, and Switzerland. Specifically, we seek to identify trauma- and patient-related factors influencing the timing of surgery and to assess their correlation with functional impairment at hospital discharge. For this purpose, we utilized the TraumaRegister DGU^®^, which has recorded data from severely injured patients from the site of the accident through discharge from acute care hospitals over many years.

## Methods

### *TraumaRegister DGU*^®^

The TraumaRegister DGU^®^ (TR-DGU) of the German Trauma Society (Deutsche Gesellschaft für Unfallchirurgie e.V., DGU) was founded in 1993. This multicenter database provides pseudonymized information about severely injured patients. Data are collected prospectively starting at the site of the accident until discharge from hospital at the following times: A) pre-hospital phase, B) emergency room and initial surgery, C) intensive care unit and D) discharge. The documentation includes detailed subject-level information on demographics, injury patterns, comorbidities, pre- and in-hospital management, treatment and care in the intensive care unit (ICU), relevant laboratory findings, transfusion and outcome data. The inclusion criteria in the registry are admission to the hospital via the emergency room with subsequent care in the ICU or admission to the hospital with vital signs still being present with death reported before admission to the ICU. Participation in TraumaRegister DGU^®^ is voluntary. For hospitals associated with TraumaNetzwerk DGU^®^, however, the entry of at least a basic data set is obligatory for reasons of quality assurance. Compared to the standard form, the basic dataset does not contain all surgical procedures.

The study utilized a retrospective cohort design, selecting patients from the TR-DGU on the basis of key variables related to spine trauma, persistent neurological deficits, timing of spine surgery and functional impairment. This study aimed to investigate associations between these variables with a particular focus on the timing of surgery and functional impairment. The study was conducted in accordance with STrengthening the Reporting of OBservational studies in Epidemiology (STROBE; www.strobe-statement.org) the Declaration of Helsinki, Good Clinical Practice and applicable regulatory requirements. The scientific data analysis of this study was approved according to a peer-review process and it is in line with the publication guidelines of the TR-DGU and registered as TR-DGU project ID 2021–006.

### Inclusion and exclusion criteria

The selection of patients was based on specific inclusion and exclusion criteria. The inclusion criteria consisted of enrollment in the TR-DGU from 2008 to 2022, spine trauma with non-transient neurological deficits, at least 16 years of age, whereas the exclusion criteria included patients admitted to hospitals outside Germany, Austria and Switzerland, missing data based on the Glasgow Outcome Scale (GOS) and no available information regarding the date of spine surgery. For the analysis of variables related to the timing of surgery only patients, who actually underwent spine surgery, were included. To avoid confusion with the American Spinal Injury Association (ASIA) Impairment Scale commonly referred to as the AIS [15], the Abbreviated Injury Scale is abbreviated here as AbbIS.

### Variables

For the spine surgery timing analysis patients were categorized into an *early surgery group* (day 0 = within the calendar day of admission) or a *late surgery group* (day after the calendar day of admission or later). Only cases with completed standard documentation forms containing specific respective information were evaluated. The severity of SCI is coded in the TR-DGU using the 6-point ordinal-scaled AbbIS severity grading (higher scores are worse) [8]. In the TR-DGU incomplete SCI is classified as AbbIS 4 and complete SCI with a lesion level below C3 or incomplete SCI in combination with laceration of the spinal cord is classified as AbbIS 5 (Table 1). High cervical complete SCI (C1-C3) is classified as AbbIS 6. In the case of multilevel SCI according to spine imaging, the higher AbbIS is chosen. In the case of equally severe SCI the more rostral injury was recorded. All AbbIS codes reflecting either incomplete or complete SCI are shown in Table 1. All injuries beyond the spine were also classified and recorded according to the AbbIS [8].

**Table 1:** Abbreviated Injury Scale (AbbIS) codes for spinal cord injuries (the number after the period denotes the level of severity (4 to 6)

AbbIS Code	Description
640210.4	Cervical spinal cord, contusion (incomplete)
640220.5	Cervical spinal cord, C4 or caudal, contusion (complete)
640240.5	Cervical spinal cord, laceration (incomplete)
640260.5	Cervical spinal cord, C4 or caudal, laceration (complete)
640229.6	Cervical spinal cord, C1-C3, contusion (complete)
640269.6	Cervical spinal cord, C1-C3, laceration (complete)
640410.4	Thoracic spinal cord, contusion (incomplete)
640420.5	Thoracic spinal cord, contusion (complete)
640440.5	Thoracic spinal cord, laceration (incomplete)
640460.5	Thoracic spinal cord, laceration (complete)
640610.4	Lumbar spinal cord, contusion (incomplete)
640620.5	Lumbar spinal cord, contusion (complete)
640640.5	Lumbar spinal cord, laceration (incomplete)
640660.5	Lumbar spinal cord, laceration (complete)

As a comprehensive measure of the functional deficits following trauma we used the GOS in all surviving patients, which consists of five categories: deceased, minimally responsive, severely disabled, moderately disabled or good recovery. For the multivariate analysis, patients were dichotomized into either *moderate functional impairment* (moderately disabled or good recovery) or *severe functional impairment* (minimally responsive, severely disabled, deceased) groups.

The key variables of age, sex, injury severity, level of injury (cervical, thoracic, lumbar), high cervical (C1-C3) injury (yes/no), signs of hemorrhage (shock with systolic blood pressure B ≤ 90 mmHg, blood transfusion before ICU admission, coagulopathy (Partial Tromboplastin Time – PTT ≥ 40 s, or INR ≥ 1.4), administration of catecholaminergic drugs in the emergency room), traumatic brain injury (TBI; AbbIS grade), relevant comorbidities according to the American Society of Anesthesiologists (ASA) risk classification, mechanical ventilation in the ICU (yes/no), trauma center level of care and timing of spine surgery (day 0, or later) were extracted from the TR-DGU. The Revised Injury Severity Classification score, version II (RISC II) was used to estimate the risk of death based on early predictors available on admission. It was developed and validated using TR-DGU data [13]. The following weighted risk factors were used to calculate the RISC II: worst and second worst injury, head injury, age, sex, motor and pupil response, blunt/penetrating trauma, blood pressure, hemoglobin, base deficit, coagulation, and cardiac arrest.

### Statistical analysis

Univariate analysis was performed with the number of patients and percentage in case of categorical data, and the mean with standard deviation (SD) for metric data. The skewed data are presented as median with quartiles. Formal statistical testing was largely avoided since small differences (approximately 2–3% in the case of categorical data) would become ‘significant’ although not clinically relevant. Adjusted effects were calculated using logistic regression analysis with various dependent variables (early surgery, moderate functional impairment). The results are presented as odds ratios (OR) with 95% confidence intervals (CI). The coefficient and the respective *p *value are also provided. All calculations were performed with SPSS statistical software (version 28; IBM Inc., Armonk, NY, USA). The regression analysis aimed to incorporate specific information about the type of surgical procedure as well as the time point of surgery.

## Results

### Patients

**Fig. 1:**
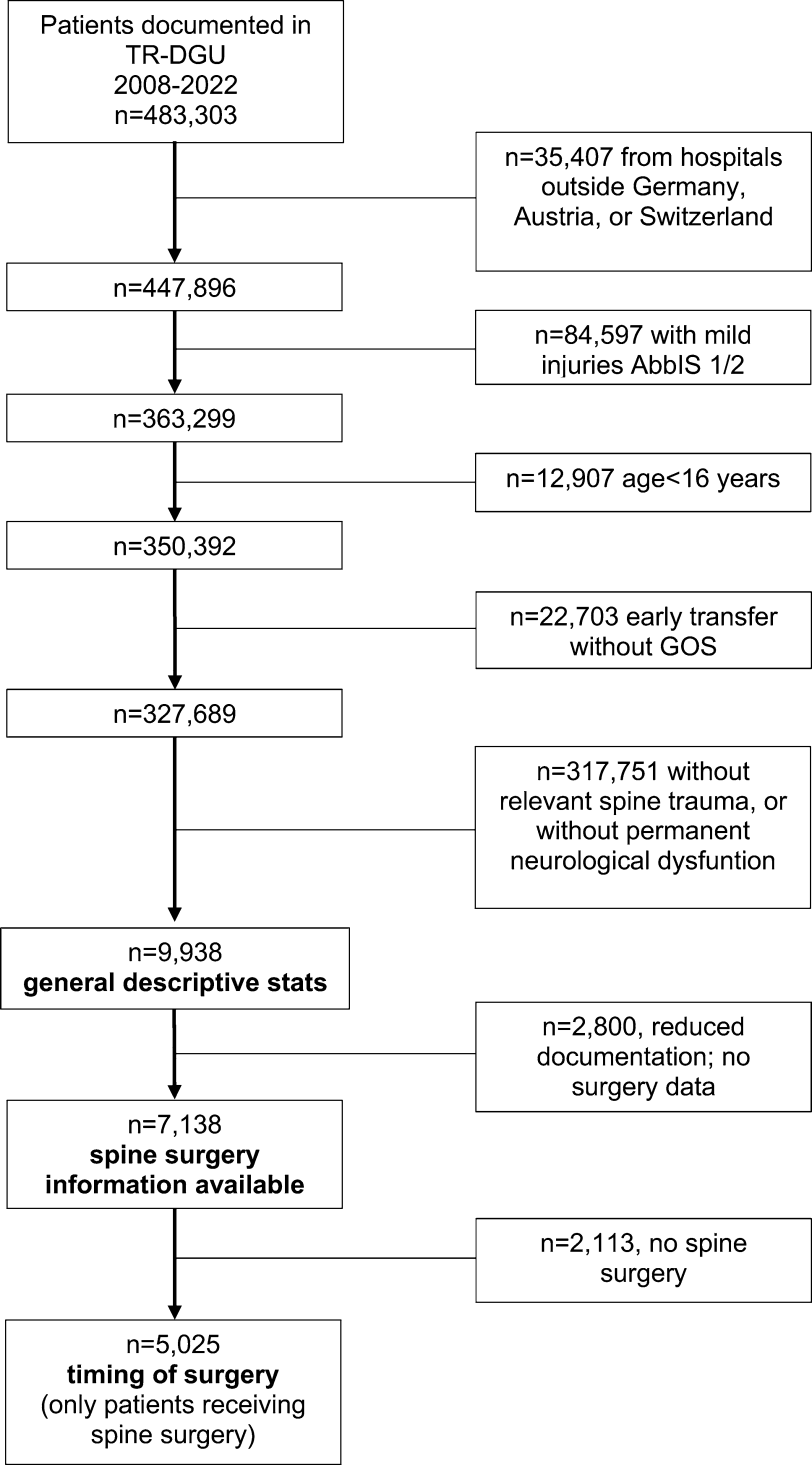
Flow chart illustrating selection of patients included in the present analysis

**Fig. 2:**
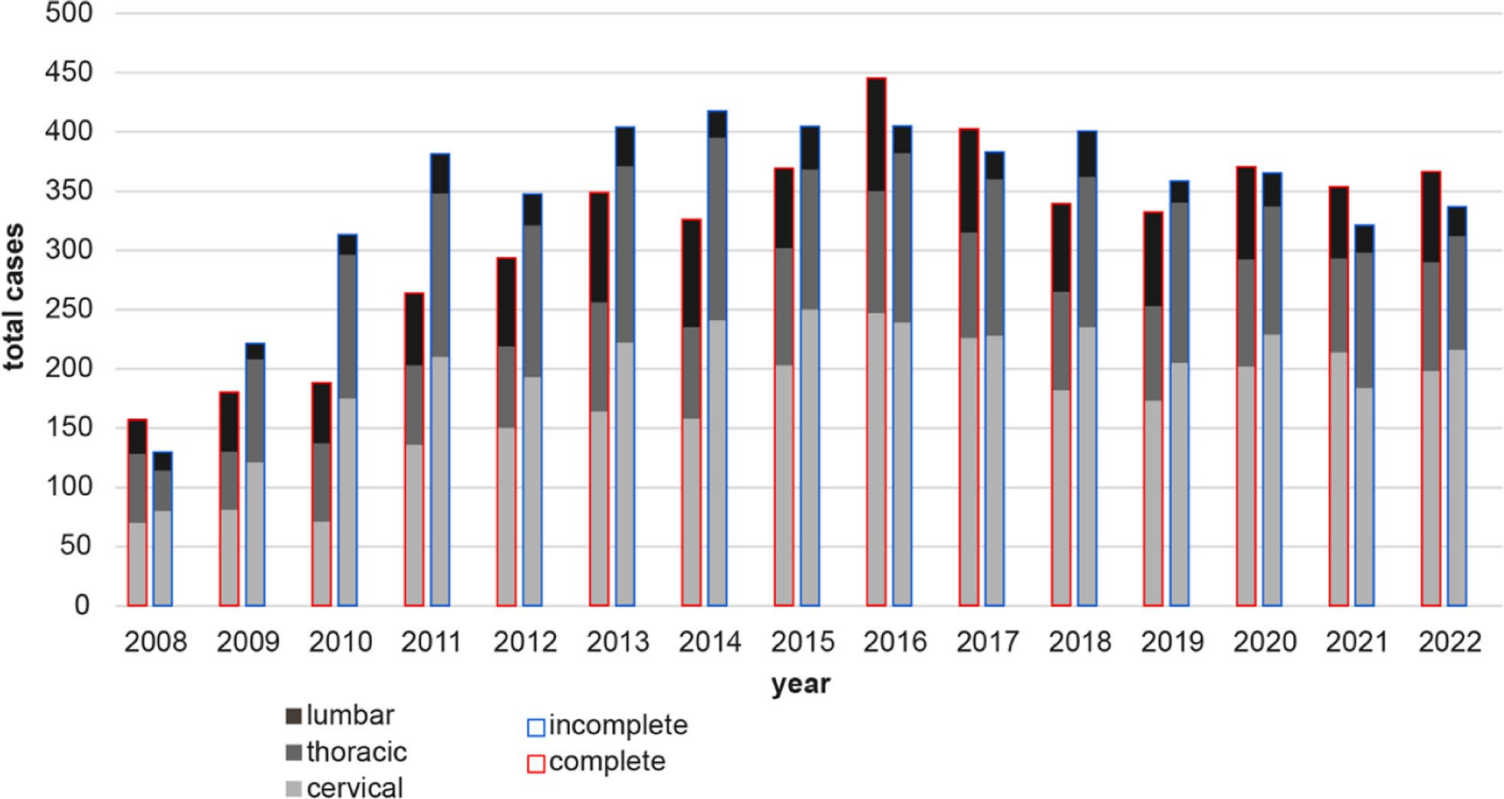
Traumatic SCI cases recorded in the TR-DGU from 2008 to 2022. Absolute numbers of incomplete (blue frame) and complete (according to Abbreviated Injury Scale—AbbIS; red frame) SCI patients per year. Injury level—cervical, thoracic, lumbar—are highlighted in different shades of gray

The database search (spine trauma AND persistent neurological dysfunction) revealed that n = 9,938 eligible patients were documented between 2008 and 2022 (Fig. 1). This cohort formed the basis for *general descriptive statistics*: traumatic SCI at cervical level represented the predominant injury level, followed by thoracic and lumbar SCI (Fig. 2). Slightly more patients suffered from complete SCI (n = 5225) than from incomplete SCI (n = 4713). A total of 1,809 (18.2%) SCI patients died during acute care with a mortality rate of 25.7% in complete and 9.9% in incomplete SCI patients. Out of all patients with a high cervical (C1-C3) SCI, 71.6% did not survive in the acute care hospital. The 9,938 patients were treated in 392 different hospitals, of which 138 (35%) were supraregional trauma centers.

The *general descriptive statistics* cohort (n = 9938 patients) included SCI patients treated in regional hospitals, where documentation was limited to a basic dataset. These smaller hospitals were granted an exception to reduce documentation workload and to facilitate participation in the TraumaRegister DGU^®^. In these cases, no details regarding surgical interventions were recorded. Based on this, 2800 patients were excluded for further evaluation. The remaining 7,138 patients, which were documented using the standard form collecting information on surgical interventions, were analyzed in the *spine surgery information available* cohort (Tables 2 and 3). Variables associated with functional impairment were also analyzed in this cohort. Of these, 2113 patients did not undergo spine surgery. Consequently, the *timing of surgery* analysis was based on 5025 patients (Fig. 1). Key characteristics of patients were not different in the three analyzed cohorts (*general descriptive statistics, spine surgery information available, timing of surgery;* Table 4) and also mirror respective features reported in the SCI focused European Multicenter Study about Spinal Cord Injury (EMSCI) [5].

**Table 2:** Surgery data for patients with incomplete and complete SCI and surgery information available (*spine surgery information available* cohort, n = 7138)

Spine surgery		Incomplete SCI	Complete SCI	Total
n^1^		3387	3751	7138
No surgery received	n (%)	934 (27.6%)	1179 (31.4%)	2113 (29.6%)
Surgery received^1^	n (%)	2453 (72.4%)	2572 (68.6%)	5025 (70.4%)
In case of surgery - on day of admission (day 0) - day 1 or later	n (%) n (%)	1643 (67.0%) 810 (33.0%)	1839 (71.5%) 733 (28.5%)	3677 (83.1%))
Early laminectomy^2^ before transfer to ICU	n (%)	680 of 1861 (36.5%)	710 of 1842 (38.5%)	1390 of 3703 (37.5%)
Time until laminectomy^2^	min	96 (77–110)	91 (69–108)	94 (72–110)

ICU, intensive care unit; SCI, spinal cord injury

^1^Timing of surgery cohort

^2^Respective information available since 2015

**Table 3:** Patient characteristics in the *spine surgery information available* cohort (n = 7138)

Spine surgery		‘Early’ Day 0	‘Late’ Day 1 or later	No spine surgery
n		3482	1543	2113
Age	mean (SD)	49.9 (20.3)	54.7 (20.3)	55.1 (21.1)
Injury Severity Score	mean (SD)	32.2 (12.2)	36.3 (16.3)	41.3 (20.0)
Injuries (AbbIS 3 +) beyond spine	n (%)	1763 (50.6%)	869 (56.3%)	1315 (62.2%)
Isolated spine trauma	n (%)	1100 (31.6%)	432 (28.0%)	545 (25.8%)
Complete SCI	n (%)	1839 (52.8%)	733 (47.5%)	1179 (55.8%)
Coagulopathy	n (%)	358 (10.7%)	308 (21.2%)	460 (24.9%)
Blood transfusion	n (%)	635 (18.2%)	251 (16.3%)	311 (14.9%)
Risk of death based on RISC II	n (%)	13.9%	25.0%	38.7%
Died in hospital	n (%)	281 (8.1%)	165 (10.7%)	739 (35.0%)
Died within 24 h	n (%)	39 (1.1%)	27 (1.7%)	384 (18.2%)
Severe traumatic brain injury (AbbIS 5/6)	n (%)	86 (5.2%)	104 (6.7%)	257 (12.2%)
Catecholamines upon admission	n (%)	907 (26.0%)	479 (31.0%)	781 (37.0%)
Shock (systolic B* p* < = 90)	n (%)	686 (19.7%)	407 (26.4%)	660 (31.2%)

AbbIS, Abbreviated Injury Scale; SCI spinal cord injury

**Table 4:** Key patient characteristics across different cohorts

	Descriptive stats cohort	Spine surgery information available cohort	Timing of surgery cohort	EMSCI cohort [5]
n	9938	7138	5025	4601
Age (mean)	52.9	52.5	51.4	47.2
Male (%)	76.0	76.1	76.9	77
Complete SCI (%)	52.6	52.5	51.2	51.5
Cervical injury level (%)	55.4	55.1	52.2	53.9

SCI, spinal cord injury; EMSCI, European Multicenter Study about Spinal Cord Injury

### Variables associated with timing of surgery

**Fig. 3:**
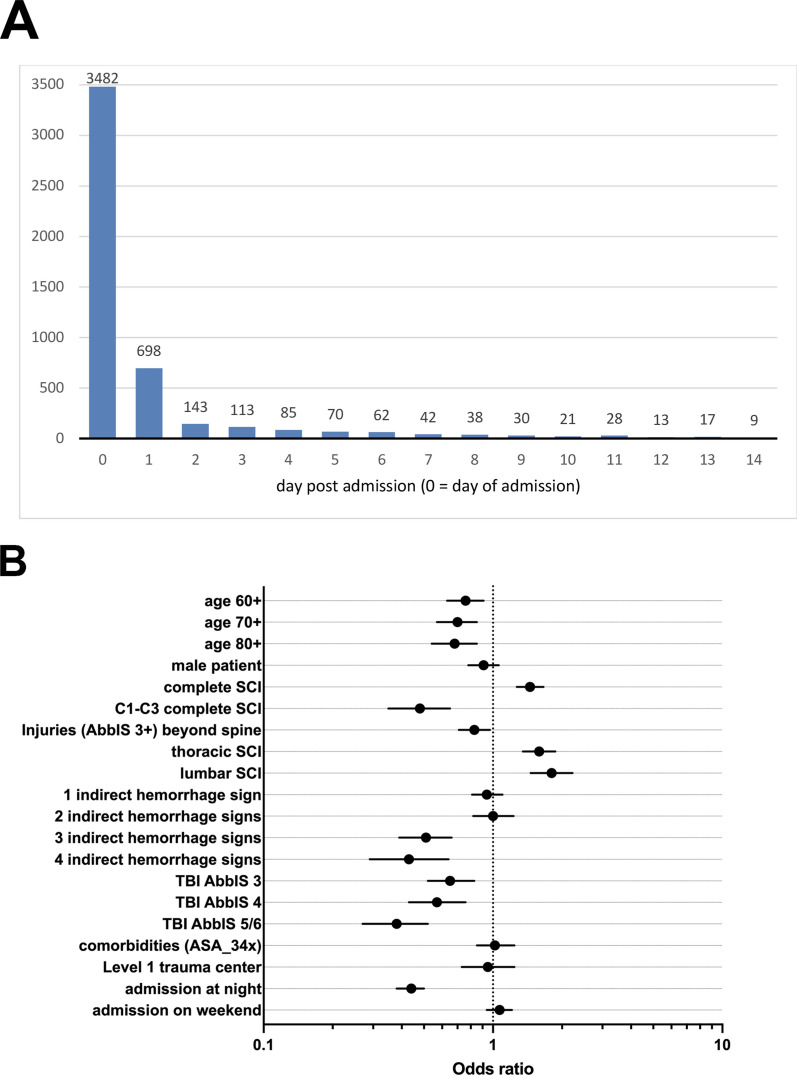
Timing of spine surgery. **A** Distribution of spine surgeries over time referenced to the day of admission. **B** Multivariate logistic regression analysis of trauma and patient related factors associated with timing of spine surgery in *the timing of surgery* cohort (only patients who received spine surgery were included). Odds ratio (OR) is shown for early spine surgery. Abbreviations: SCI, spinal cord injury; AbbIS, Abbreviated Injury Scale; TBI, traumatic brain injury

In the *spine surgery information available* cohort (Table 3), 5025 patients actually received either early (day of admission) or late spine surgery (day after admission or later). Of these, 69% underwent early spine surgery (Fig. 3A). Considering that a subset of patients in the category “late spine surgery” were still within the 24 h time frame, the overall proportion of patients within the 24 h time frame was even higher than 69%.

In a multivariate logistic regression analysis (dependent variable: late surgery versus early surgery) the following variables were independently associated with late surgery: higher age (60 years and older), high cervical injury (C1-3) compared to all other levels of SCI, relevant injuries other than spine (AbbIS ≥ 3), three or more indirect signs of hemorrhage (such as blood transfusion, coagulopathy, positive shock index, and catecholamine administration) and TBI (f. 3B, Table 5). The following variables were independently associated with early surgery: complete SCI, thoracic or lumbar level of SCI. Sex, comorbidities prior to trauma (ASA grades 3 and 4) and admission to a level 1 trauma center did not appear to play a role in this respect (Fig. 3B).

**Table 5:** Results of logistic regression analysis in 5,025 operated patients (*timing of surgery* cohort), with early spine surgery (day 0, prevalence 69.3%) as the dependent variable. Odds ratios (OR) > 1.00 are associated with early surgery, whereas factors with OR < 1.00 are associated with later surgery

Variable	*p *value	OR	95% CI	
Age (reference: < 60)	< .001			
60–69	.004	0.76	0.63	0.91
70–79	< .001	0.70	0.57	0.85
80 +	< .001	0.68	0.54	0.85
Male patient	.21	0.91	0.78	1.06
Complete SCI	< .001	1.45	1.27	1.66
C1-C3 SCI	< .001	0.48	0.35	0.65
Relevant injuries beyond the spine (AbbIS 3 +)	.022	0.83	0.71	0.97
Level of injury (reference: cervical)	< .001			
Thoracic	< .001	1.59	1.35	1.87
Lumbar	< .001	1.80	1.46	2.22
Indirect bleeding signs (reference: none)	< .001			
1	.440	0.94	0.81	1.10
2	1.00	1.00	0.82	1.23
3	< .001	0.51	0.39	0.66
4	< .001	0.43	0.29	0.64
TBI_group (reference: AbbIS 0–2)	< .001			
AbbIS 3	< .001	0.65	0.52	0.83
AbbIS 4	< .001	0.57	0.43	0.76
AbbIS 5/6	< .001	0.38	0.27	0.52
ASA_3 or 4	.81	1.02	0.85	1.24
Level 1 trauma center	0.71	0.95	0.73	1.24
Constant	< .001	3.61		

OR, odds ratio; AbbIS, Abbreviated Injury Scale; SCI, spinal cord injury; TBI, traumatic brain injury; ASA, American Society of Anesthesiologists risk classification

Patients who did not undergo spine surgery represented the most severely affected SCI patients. Compared with patients who underwent early or late spine surgery, 62.2% of those who did not undergo surgery had the highest rate of additional trauma (AbbIS greater than 3) in comparison to patients receiving early or late spine surgery showing in 50.6% and 56.3% additional trauma, respectively. Accordingly, a greater injury severity score (mean = 41.3) was found in patients not receiving spine surgery compared to patients receiving early or late spine surgery (mean = 32.2 and mean = 36.3, respectively).

Patients, who did not undergo spine surgery, were more likely to suffer from complete SCI (55.8% vs. 52.8% and 47.5% receiving early or late spine surgery, respectively) and showed more pronounced signs of shock and coagulopathy than patients, who received a spine surgical intervention at any time point beyond the day of admission (Table 3). In contrast, those patients who underwent early spine surgery tended to be younger (49.9 vs. 54.7 and 55.1 mean years of age in patients who underwent late surgery or no surgery, respectively). Patients who underwent early spine surgery were more likely to have an isolated spine trauma (31.6% vs. 28% and 25.8% of patients who received late surgery or no surgery, respectively). The majority of patients, who died during their stay in the acute care hospital (1635 patients; 18.2% of patients recorded in the standard form), did not undergo spine surgery (1123 patients; 68% of all patients, who died during the acute care stay).

### Variables associated with functional impairment

**Fig. 4:**
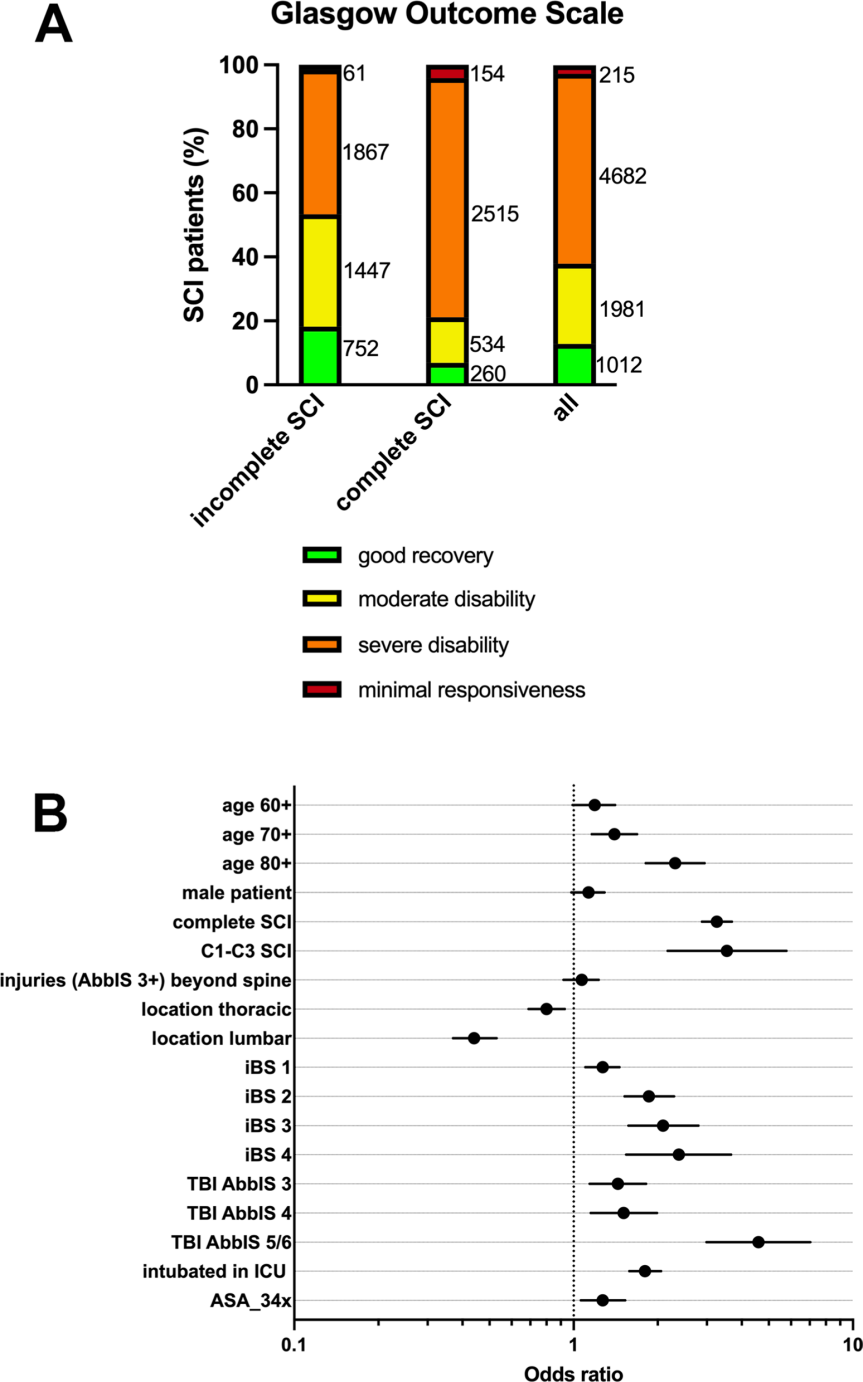
Factors associated with severe functional impairment according to the GOS. **A **Within the incomplete, complete SCI and the total SCI cohort the percentage of SCI patients with the different GOS grades, respectively are depicted. Absolute numbers are added to each column. **B** Multivariate logistic regression analysis of trauma and patient related factors associated with severe functional impairment (minimal responsiveness and severe disability according to GOS) in the *functional impairment analysis* cohort (only surviving patients without severe TBI were analyzed). Abbreviations: SCI, spinal cord injury; AbbIS, abbreviated injury scale; iBS, indirect bleeding sign; ICU, intensive care unit; ASA, American Society of Anesthesiologists risk classification; OR, operation room; TBI, traumatic brain injury; GOS, Glasgow Outcome Scale

The only scale recorded within the TR-DGU, which reflects rather coarsly functional impairment, is the Glasgow Outcome Scale (GOS) at the time of discharge, which was considered in this study as a comprehensive measure of functional impairment rather than a valid outcome parameter. Accordingly, the categories “minimally responsive”, “severely disabled” or “deceased” were defined as *severe functional impairment*, whereas “moderately disabled” or “good recovery” were considered as *moderate functional impairment*. Only a minority of SCI patients were classified as being minimally responsive, which likely refers to concomitant TBI (Fig. 4A).

Looking at the cohort of patients (with or without spine surgery), for whom spine surgery information was available, the following trauma related variables were associated with severe impairment (Fig. 4B, Table 6): patient age > 70, complete SCI, high cervical (C1-3) SCI, 1–4 signs of indirect hemorrhage, TBI and intubation in the ICU. The thoracic/lumbar level of spine injury was associated with a more moderate functional impairment. Injuries beyond the spine (AbbIS ≥ 3) and sex were not significantly associated with functional impairment according to the GOS. The same multivariate regression analysis considering only patients undergoing spine surgery (n = 4333) yielded similar results in respect to the correlation of trauma related variables with functional impairment, however age and concomitant disease correlations were not significantly different (Suppl. Table. 1).

**Table 6:** Results of logistic regression analysis in 6627 patients (*spine surgery information available* cohort; 511 had to be excluded due to missing values) with relevant spinal injuries. The dependent variable was severe functional impairment (deceased, minimally responsive or severely disabled according to the GOS; prevalence 62.3%). OR > 1.00 favor severe functional impairment, whereas OR < 1.00 favor moderate functional impairment

Variable	*p* value	OR	95% CI	
Age (reference: < 60)	< .001			
60 +	.052	1.19	0.99	1.41
70 +	< .001	1.40	1.16	1.69
80 +	< .001	2.31	1.81	2.95
Male patient	.089	1.13	.98	1.29
Complete SCI	< .001	3.26	2.88	3.69
C1-C3 SCI	< .001	3.54	2.17	5.78
Injuries beyond spine (AbbIS 3 +)	.390	1.07	0.92	1.23
Location (reference: cervical))	< .001			
Thoracic	.004	.80	0.69	0.93
Lumbar	< .001	.44	0.37	0.53
Indirect bleeding signs (reference: none)		< .001		
1	.001	1.27	1.10	1.46
2	< .001	1.86	1.52	2.29
3	< .001	2.09	1.57	2.80
4	< .001	2.38	1.54	3.67
TBI_group (reference: AbbIS 0–2)	< .001			
AbbIS 3	.002	1.44	1.14	1.82
AbbIS 4	.003	1.51	1.15	1.99
AbbIS 5/6	< .001	4.59	2.99	7.04
Mechanical ventilation in ICU	< .001	1.80	1.58	2.06
ASA_34x	.011	1.27	1.06	1.53

OR, odds ratio; SCI, spinal cord injury; AbbIS, Abbreviated Injury Scale; TBI, traumatic brain injury; ICU, intensive care unit; ASA American Society of Anesthesiologists risk classification

Nagelkerke’s R^2^ = 0.207

## Discussion

Analyses of a large cohort of prospectively recorded acute traumatic spinal cord injury (SCI) patients across acute care hospitals in Germany, Austria, and Switzerland contributing to the DGU registry revealed that the majority underwent spine surgery shortly after injury. A significant proportion received surgical intervention on the day of admission, consistent with current clinical guidelines [6]. Most trauma- and patient-related factors associated with the timing of surgery also explained the degree of functional impairment, as measured by the Glasgow Outcome Scale (GOS).

In our study, 69% of patients underwent spine surgery on the day of admission, underscoring a strong emphasis on early surgical intervention. Since “day 0” refers to the full day of admission (0–24 h), and most admissions occurred during the second half of the day, the peak time window for surgery likely fell within the 0–12-h post-injury period. This figure is comparable to findings from a retrospective analysis in the US and Canada (2010–2016, thoracolumbar injuries only), where approximately 60% of operated patients (n = 3948) received surgery within 24 h post-injury [4]. In contrast, a smaller cohort in Norway (2015–2022, cervical injuries only; n = 243) reported that 47% of patients received surgery within 24 h [1]. At the other extreme, a large Chinese retrospective analysis (2013–2018, all injury levels; n = 10,053) showed that only 2.8% of patients underwent surgery within the first 24 h [26]. These international differences likely reflect variability in healthcare infrastructure, trauma protocols, surgical resources, and hospital transfer systems. While high rates of early surgery in our cohort and in North America align with guideline recommendations for timely decompression [6], the significantly lower rates in China may point to systemic barriers such as limited access to spine surgeons, inter-hospital transfer delays, or differing clinical decision-making processes. Understanding these disparities is critical for improving global access to timely spine surgery and ensuring optimal care for SCI patients regardless of geographic location.

Before 2020, the documentation regarding surgical interventions in the TraumaRegister DGU^®^ allowed free text input and thus precludes a systematic respective analysis, which would have allowed more detailed insights into the type of spine surgical intervention (e.g. spine decompression versus spine stabilization). From 2020 on, surgical interventions could be selected from a small list of procedures for different types of injuries and are thus available for systematic analysis. We chose to not present respective data, since they cover only the years from 2020 until now and thus do not match the time frame of the spine surgery analysis in the present study. To get a general idea, between 2020 and 2024 laminectomy was the most frequent spine surgical procedure in over 50% of patients receiving early or late spine surgery.

Only a few studies have investigated variables influencing the timing and decision-making for spine surgery [2, 12]. Consistent with our findings, Kopp et al. reported that older age (≥ 75 years) was associated with a prolonged surgery interval (22.8 h), whereas younger patients (≤ 44 years) underwent surgery sooner (6.6 h). In our cohort, complete SCI—as coded by the AbbIS classification—was associated with a higher likelihood of early spine surgery (on the day of admission) compared to incomplete SCI, aligning with Canadian data indicating that early surgery (< 24 h) is more commonly performed in patients with complete injuries (AIS grade A) [9]. Conversely, a survey of Dutch spine surgeons suggested a preference for early surgery in patients with incomplete SCI [20]. It is well established that older age is associated with poorer functional outcomes following SCI [25]. Given that older age is also associated with delayed surgical intervention, this may partially explain the inferior outcomes seen in previous studies examining surgical timing.

In our study, patients with C1–C3 cervical spine trauma underwent spine surgery later (beyond the day of admission). According to the literature, patients with translational (predominantly subaxial) cervical spine fractures are more likely to receive surgery earlier—within 24 h [1]. Since translational fractures in the upper cervical spine (C0–C2) are rare [19], this may account for the delayed surgery observed in this subgroup. Regarding TBI and its association with delayed spine surgery, our findings align with a large U.S. registry study, which reported a delay in surgical timing for patients with concomitant TBI—from around 20 to 24 h post-injury [3]. That study speculated that preoperative requirements—such as neurological examination, obtaining informed consent, and collecting past medical history (e.g., metal implants, anticoagulant use)—may contribute to surgical delays. Based on our multivariate analysis, additional factors may also play a role in postponing spine surgery. The presence of other relevant injuries and signs of hemorrhage, both correlated with delayed intervention, suggest that more urgent non-spinal procedures may have taken precedence. This likely applies to TBI as well: as TBI severity increased, the probability of delayed spine surgery rose (as reflected by the decreasing odds ratio in more severe TBI cases). Interventions to e.g. reduce intracranial pressure due to hydrocephalus, edema, or hemorrhage may have been prioritized.

To date, few studies have examined correlations between admission blood pressure and timing of surgery. Our data clearly show that patients presenting with signs of circulatory instability (systolic blood pressure < 90 mmHg or requiring catecholamines) were more likely to experience surgical delays. Consistent with existing evidence, several studies have found that intraoperative hypotension is associated with unfavorable neurological outcomes, leading to recommendations that mean arterial pressure (MAP) be maintained at a minimum of 85 mmHg [17, 22]. However, this association was not replicated in a recent study of 319 SCI patients [18], where no correlation was found between emergency room hypotension and functional outcomes (FIM) in a subacute rehabilitation setting. Since detailed hemodynamic data were not provided, it is difficult to interpret this discrepancy. Notably, the Shea study analyzed a selected patient group transferred to specialized rehabilitation centers, whereas our registry-based study included all patients with acute SCI, regardless of their subsequent care pathway. This broader inclusion may explain why we found a correlation between shock index and severe functional impairment.

The findings reported here—alongside previous registry-based data [10]—underscore the importance of accounting for confounding factors when interpreting studies that evaluate early surgery as an independent predictor of functional outcome [7]. A recent publication emphasized the role of confounders such as early outcome assessments and ceiling effects, both of which may bias the interpretation of primary and secondary endpoints [11]. One limitation of our study is the lack of a robust long-term outcome measure. While the GOS is included in the TraumaRegister DGU^®^, its limited granularity and early time point (discharge from acute care) do not allow to perform a meaningful outcome analysis. In contrast, many prior studies that linked early surgery with superior outcomes employed detailed neurological assessments (e.g., ISNCSCI motor scores, AIS grade) performed 6–12 months post-injury.

The number of patients with traumatic SCI included in the registry increased between 2008 and 2013, stabilizing thereafter at an annual incidence of approximately 700–800 cases. This rise likely reflects an increasing number of trauma centers submitting data to maintain network certification. Since 2013, roughly 90% of trauma centers have contributed to the TraumaRegister DGU^®^. However, it is important to note that the registry may underestimate the true incidence of SCI, as it includes only patients admitted to intensive care units. A recent study using ICD-10 data from the German Federal Bureau of Statistics estimated an annual incidence of approximately 1300 cases between 2013 and 2020 [14].

The high exclusion rate of patients with spine trauma documented in the TraumaRegister DGU^®^ bears the risk of selection bias and thus represents a clear limitation of the present study. However, comparison with EMSCI, an international network of SCI centers examining neurological recovery within the first year after injury [5], yields similar core characteristics (age, gender, injury level, injury severity).

## Conclusions

This large registry-based analysis shows that factors such as older age, circulatory instability, high cervical SCI, and severe concomitant trauma are associated with both delayed spine surgery and severe functional impairment. These overlapping associations highlight the difficulty of disentangling the effects of surgical timing from underlying patient and injury characteristics. While early surgery was common in our cohort – in line with best practice guidelines—and may be linked to better outcomes, these findings caution against interpreting timing as an independent predictor without accounting for significant confounding factors. To clarify the true impact of early surgical intervention in SCI, future research must rely on prospective, multicenter studies using detailed, long-term functional assessments together with rigorous recording of confounders in the early phase. Only through such designs integrated into patient registries such as the TraumaRegister DGU^®^ and the European Multicenter Study about SCI (EMSCI) [5], covering the early and later phase after injury respectively, can we accurately inform clinical guidelines and improve patient care.

## Supplementary Information

Additional file 1.

## Data Availability

According to the guidelines of the German Trauma Society (DGU) and the Akademie der Unfallchirurgie (AUC GmbH) who runs the registry, raw data from TR-DGU are not publicly available. However, applications for analyses can be forwarded to AUC GmbH.

## Abbreviations

AbbIS: Abbreviated injury scale
AIS: ASIA impairment scale
ASA: American Society of Anesthesiologists risk classification
ASIA: American Spinal Injury Association
FIM: Functional independence measure
GOS: Glasgow outcome scale
ICU: Intensive care unit
ISNCSCI: International standards for neurological classification of spinal cord injury
OR: Odds ratio
PTT: Partial tromboplastin time
RISC II: Revised injury severity classification score, version II
SCI: Spinal cord injury
SCIM: Spinal cord independence measure
STROBE: Strengthening the reporting of observational studies in epidemiology
TBI: Traumatic brain injury
TR-DGU: TraumaRegister DGU®

## References

[1] Aarhus, M., Mirzamohammadi, J., Ronning, P. A., Strom, M., Glott, T., Rizvi, S. A. M., Biernat, D., Olstorn, H., Rydning, P. N. F., Stenset, V. T. V., Naess, P. A., Gaarder, C., Brommeland, T., Linnerud, H., & Helseth, E. (2024). Time from injury to acute surgery for patients with traumatic cervical spinal cord injury in South-East Norway. *Frontiers in Neurology,**15*, 1420530. 10.3389/fneur.2024.1420530 38978812 10.3389/fneur.2024.1420530 PMC11228170

[2] Ahn, H., Bailey, C. S., Rivers, C. S., Noonan, V. K., Tsai, E. C., Fourney, D. R., Attabib, N., Kwon, B. K., Christie, S. D., Fehlings, M. G., Finkelstein, J., Hurlbert, R. J., Townson, A., Parent, S., Drew, B., Chen, J., Dvorak, M. F., Rick Hansen Spinal Cord Injury Registry N. (2015). Effect of older age on treatment decisions and outcomes among patients with traumatic spinal cord injury. *Canadian Medical Association Journal,**187*(12), 873–880. 10.1503/cmaj.150085 26149702 10.1503/cmaj.150085 PMC4562825

[3] Azad, T. D., Raj, D., Ran, K. R., Vattipally, V. N., Warman, A., Raad, M., Williams, J. R., Lubelski, D., Haut, E. R., Suarez, J. I., Bydon, A., Witham, T. F., Witiw, C. D., Theodore, N., & Byrne, J. P. (2024). Concomitant traumatic brain injury delays surgery in patients with traumatic spinal cord injury. *Neurosurgery*. 10.1227/neu.0000000000002816 38197654 10.1227/neu.0000000000002816

[4] Badhiwala, J. H., Wilson, J. R., Witiw, C. D., Harrop, J. S., Vaccaro, A. R., Aarabi, B., Grossman, R. G., Geisler, F. H., & Fehlings, M. G. (2021). The influence of timing of surgical decompression for acute spinal cord injury: A pooled analysis of individual patient data. *The Lancet Neurology,**20*(2), 117–126. 10.1016/S1474-4422(20)30406-3 33357514 10.1016/S1474-4422(20)30406-3

[5] Bourguignon, L., Tong, B., Geisler, F., Schubert, M., Rohrich, F., Saur, M., Weidner, N., Rupp, R., Kalke, Y. B., Abel, R., Maier, D., Grassner, L., Chhabra, H. S., Liebscher, T., Cragg, J. J., group, E. s., Kramer, J., Curt, A., & Jutzeler, C. R. (2022). International surveillance study in acute spinal cord injury confirms viability of multinational clinical trials. *BMC Medicine,**20*(1), Article 225. 10.1186/s12916-022-02395-0 35705947 10.1186/s12916-022-02395-0 PMC9202190

[6] Cryns, N., Himmelhaus, S., Irrgang, S., Ernst, M., Weidner, N., & Scheel-Sailer, A. (2025). Clinical practice guideline: the diagnosis and treatment of acute spinal cord injury. *Deutsches Ärzteblatt International*. 10.3238/arztebl.m2025.0034. Forthcoming. 40073287 10.3238/arztebl.m2025.0034 PMC12516205

[7] Fehlings, M. G., Hachem, L. D., Tetreault, L. A., Skelly, A. C., Dettori, J. R., Brodt, E. D., Stabler-Morris, S., Redick, B. J., Evaniew, N., Martin, A. R., Davies, B., Farahbakhsh, F., Guest, J. D., Graves, D., Korupolu, R., McKenna, S. L., & Kwon, B. K. (2024). Timing of decompressive surgery in patients with acute spinal cord injury: Systematic review update. *Global Spine Journal,**14*(3_suppl), 38S-57S. 10.1177/21925682231197404 38526929 10.1177/21925682231197404 PMC10964893

[8] Genarelli, T. A., & Wodzin, E. (2008). Abbreviated Injury Scale 2005 – Update 2008. In A. f. t. A. o. A. M. (AAAM) (Ed.).

[9] Glennie, R. A., Bailey, C. S., Tsai, E. C., Noonan, V. K., Rivers, C. S., Fourney, D. R., Ahn, H., Kwon, B. K., Paquet, J., Drew, B., Fehlings, M. G., Attabib, N., Christie, S. D., Finkelstein, J., Hurlbert, R. J., Parent, S., & Dvorak, M. F. (2017). An analysis of ideal and actual time to surgery after traumatic spinal cord injury in Canada. *Spinal Cord,**55*(6), 618–623. 10.1038/sc.2016.177 28418395 10.1038/sc.2016.177

[10] Hax, J., Teuben, M., Halvachizadeh, S., Berk, T., Scherer, J., Jensen, K. O., Lefering, R., Pape, H. C., Sprengel, K., & TraumaRegister, D. G. U. (2023). Timing of spinal surgery in polytrauma: The relevance of injury severity, injury level and associated injuries. *Global Spine Journal*. 10.1177/21925682231216082 37963389 10.1177/21925682231216082 PMC11877677

[11] Hosman, A. J. F., Barbagallo, G., The, S. C. I. P. S. G., Popescu, E. C., van de Meent, H., Oner, F. C., De Iure, F., Bonavita, J., Kreinest, M., Lindtner, R. A., Quraishi, N. A., Thumbikat, P., Bilic, V., Reynolds, J. J., Belci, M., Borcek, A. O., Morris, S., Hoffmann, C., Signorelli, F., … van Middendorp, J. J. (2023). Neurological recovery after early versus delayed surgical decompression for acute traumatic spinal cord injury. *Bone Joint Journal,**105-B*(4), 400–411. 10.1302/0301-620X.105B4.BJJ-2022-0947.R2 36924174 10.1302/0301-620X.105B4.BJJ-2022-0947.R2

[12] Kopp, M. A., Lubstorf, T., Blex, C., Schwab, J. M., Grittner, U., Auhuber, T., Ekkernkamp, A., Niedeggen, A., Prillip, E., Hoppe, M., Ludwig, J., Kreutztrager, M., & Liebscher, T. (2022). Association of age with the timing of acute spine surgery-effects on neurological outcome after traumatic spinal cord injury. *European Spine Journal,**31*(1), 56–69. 10.1007/s00586-021-06982-2 34533643 10.1007/s00586-021-06982-2

[13] Lefering, R., Huber-Wagner, S., Nienaber, U., Maegele, M., & Bouillon, B. (2014). Update of the trauma risk adjustment model of the TraumaRegister DGU: The revised injury severity classification, version II. *Critical Care,**18*(5), Article 476. 10.1186/s13054-014-0476-2 25394596 10.1186/s13054-014-0476-2 PMC4177428

[14] Rau, Y., Schulz, A. P., Thietje, R., Matrisch, L., Frese, J., & Hirschfeld, S. (2023). Incidence of spinal cord injuries in Germany. *European Spine Journal,**32*(2), 601–607. 10.1007/s00586-022-07451-0 36371751 10.1007/s00586-022-07451-0 PMC9660155

[15] Rupp, R., Biering-Sorensen, F., Burns, S. P., Graves, D. E., Guest, J., Jones, L., Read, M. S., Rodriguez, G. M., Schuld, C., Tansey-Md, K. E., Walden, K., & Kirshblum, S. (2021). International standards for neurological classification of spinal cord injury: Revised 2019. *Topics in Spinal Cord Injury Rehabilitation,**27*(2), 1–22. 10.46292/sci2702-1 34108832 10.46292/sci2702-1 PMC8152171

[16] Seblani, M., Decherchi, P., & Brezun, J. M. (2023). Edema after CNS trauma: A focus on spinal cord injury. *International Journal of Molecular Sciences*. 10.3390/ijms24087159 37108324 10.3390/ijms24087159 PMC10138956

[17] Sharif, S., & Jazaib Ali, M. Y. (2020). Aug). Outcome prediction in spinal cord injury: Myth or reality. *World Neurosurgery,**140*, 574–590. 10.1016/j.wneu.2020.05.043 32437998 10.1016/j.wneu.2020.05.043

[18] Shea, C., Slocum, C., Goldstein, R., Roach, M. J., Griffin, R., Chen, Y., & Zafonte, R. (2022). Trauma indicators in spinal cord injury rehabilitation outcomes: A retrospective cohort analysis of the National Trauma Data Bank and National Spinal Cord Injury Database. *Archives of Physical Medicine and Rehabilitation,**103*(4), 642–6485. 10.1016/j.apmr.2021.12.001 34936887 10.1016/j.apmr.2021.12.001

[19] Strom, M., Mirzamohammadi, J., Glott, T., Brommeland, T., Linnerud, H., Ronning, P. A., Mujtaba Rizvi, S. A., Biernat, D., Arnoy Austad, T., Efskind Harr, M., Aarhus, M., & Helseth, E. (2025). Epidemiology of traumatic cervical spinal cord injury in Southeast Norway. *Neurotrauma Reports,**6*(1), 539–550. 10.1089/neur.2025.0013 40630650 10.1089/neur.2025.0013 PMC12235120

[20] Ter Wengel, P. V., Feller, R. E., Stadhouder, A., Verbaan, D., Oner, F. C., Goslings, J. C., & Vandertop, W. P. (2018). Timing of surgery in traumatic spinal cord injury: A national, multidisciplinary survey. *European Spine Journal,**27*(8), 1831–1838. 10.1007/s00586-018-5551-y 29572739 10.1007/s00586-018-5551-y

[21] Ter Wengel, P. V., Reith, F., Adegeest, C. Y., Fehlings, M. G., Kwon, B. K., Vandertop, W. P., & Oner, C. F. (2024). Under pressure - A historical vignette on surgical timing in traumatic spinal cord injury. *Brain Spine,**4*, Article 102825. 10.1016/j.bas.2024.102825 38756860 10.1016/j.bas.2024.102825 PMC11096936

[22] Torres-Espin, A., Haefeli, J., Ehsanian, R., Torres, D., Almeida, C. A., Huie, J. R., Chou, A., Morozov, D., Sanderson, N., Dirlikov, B., Suen, C. G., Nielson, J. L., Kyritsis, N., Hemmerle, D. D., Talbott, J. F., Manley, G. T., Dhall, S. S., Whetstone, W. D., Bresnahan, J. C., … Investigators, T.-S. (2021). Topological network analysis of patient similarity for precision management of acute blood pressure in spinal cord injury. *eLife*. 10.7554/eLife.68015 34783309 10.7554/eLife.68015 PMC8639149

[23] van Middendorp, J. J., Hosman, A. J., & Doi, S. A. (2013). The effects of the timing of spinal surgery after traumatic spinal cord injury: a systematic review and meta-analysis. *Journal of Neurotrauma,**30*(21), 1781–1794. 10.1089/neu.2013.2932 23815524 10.1089/neu.2013.2932

[24] Wang, T. Y., Park, C., Zhang, H., Rahimpour, S., Murphy, K. R., Goodwin, C. R., Karikari, I. O., Than, K. D., Shaffrey, C. I., Foster, N., & Abd-El-Barr, M. M. (2021). Management of acute traumatic spinal cord injury: A review of the literature. *Frontiers in Surgery,**8*, Article 698736. 10.3389/fsurg.2021.698736 34966774 10.3389/fsurg.2021.698736 PMC8710452

[25] Wirz, M., & Dietz, V. (2012). Concepts of aging with paralysis: Implications for recovery and treatment. *Handbook of Clinical Neurology,**109*, 77–84. 10.1016/B978-0-444-52137-8.00005-X 23098707 10.1016/B978-0-444-52137-8.00005-X

[26] Zhou, H., Lou, Y., Chen, L., Kang, Y., Liu, L., Cai, Z., Anderson, D. B., Wang, W., Zhang, C., Wang, J., Ning, G., Gao, Y., He, B., Ding, W., Wang, Y., Mei, W., Song, Y., Zhou, Y., Xia, M., … Feng, S. (2024). Epidemiological and clinical features, treatment status, and economic burden of traumatic spinal cord injury in China: A hospital-based retrospective study. *Neural Regeneration Research,**19*(5), 1126–1133. 10.4103/1673-5374.382257 37862218 10.4103/1673-5374.382257 PMC10749597

