# Supplementary material for: The race against time: patterns and variables of spine surgery timing in traumatic spinal cord injury: a retrospective cohort study from the TraumaRegister DGU®
Source: Neurol Res Pract. 2025 Oct 10;7(1):74. doi: 10.1186/s42466-025-00429-5 (PMC12514803; doi:10.1186/s42466-025-00429-5)
Supplement: Supplementary file 1 — Additional file 1. [file 42466_2025_429_MOESM1_ESM.docx]

**Suppl. Table 1** Results of logistic regression analysis in 4,333 patients (n=411 deceased, n=118 without information regarding GOS, n=128 with severe traumatic brain injury AIS 5/6 were excluded). The dependent variable was severe functional impairment (minimally responsive or severely disabled according to the GOS; prevalence 62.9%). OR >1.00 favor severe functional impairment , whereas OR<1.00 favor moderate functional impairment.

|  | **p value** | **OR** | **95% CI** | |
| --- | --- | --- | --- | --- |
| Age (reference: <60) | ,380 |  |  |  |
| 60+ | ,253 | 1,13 | 0,92 | 1,38 |
| 70+ | ,227 | 1,15 | 0,92 | 1,44 |
| 80+ | ,200 | 1,22 | 0,90 | 1,64 |
| Male patient | ,031 | ,708 | 1,03 | 0,88 |
| Complete SCI | 1,135 | <,001 | 3,11 | 2,69 |
| C1-C3 SCI | ,468 | ,126 | 1,60 | 0,88 |
| Injuries beyond spine (AbbIS 3+) | ,043 | ,544 | 1,04 | 0,88 |
| Location (reference: cervical)) |  | <,001 |  |  |
| thoracic | -,337 | <,001 | 0,71 | 0,60 |
| lumbar | -,878 | <,001 | 0,42 | 0,34 |
| Indirect bleeding signs (reference: none) |  | <.001 |  |  |
| 1 | ,001 | 1,32 | 1,12 | 1,56 |
| 2 | <,001 | 1,67 | 1,31 | 2,12 |
| 3 | <,001 | 1,82 | 1,31 | 2,54 |
| 4 | ,120 | 1,52 | 0,90 | 2,56 |
| TBI_group (reference: AbbIS 0-2) | <,001 |  |  |  |
| AbbIS 3 | ,002 | 1,55 | 1,18 | 2,04 |
| AbbIS 4 | ,007 | 1,59 | 1,13 | 2,23 |
| Mechanical ventilation in ICU | <,001 | 1,50 | 1,28 | 1,75 |
| ASA_34x | ,285 | 1,13 | 0,90 | 1,41 |

*Abbreviations: OR odds ratio, SCI spinal cord injury, AbbIS Abbreviated Injury Scale, TBI traumatic brain injury, ICU intensive care unit, ASA American Society of Anesthesiologists risk classification*

Nagelkerke’s R² = 0.207
